# Salvage prostate re-irradiation using high-dose-rate brachytherapy or focal stereotactic body radiotherapy for local recurrence after definitive radiation therapy

**DOI:** 10.1186/s13014-017-0789-9

**Published:** 2017-03-09

**Authors:** Aurélie Mbeutcha, Laurent Chauveinc, Pierre-Yves Bondiau, Marie-Eve Chand, Matthieu Durand, Daniel Chevallier, Jean Amiel, Daniel Lam Cham Kee, Jean-Michel Hannoun-Lévi

**Affiliations:** 1 Department of Radiation Oncology, Antoine-Lacassagne Cancer Canter, University of Nice Sophia-Antipolis, 33, avenue Valombrose, 06189 Nice Cedex 2 Nice, France; 2 Department of Urology, Hôpital Pasteur 2, Centre Hospitalier Universitaire de Nice, University of Nice Sophia-Antipolis, Nice, France; 3 Department of Radiation Oncology, Clinique Hartmann, Levallois-Perret, France

**Keywords:** Prostate cancer, Local recurrence, Salvage therapy, High-dose-rate brachytherapy, Stereotactic body radiation therapy, CyberKnife

## Abstract

**Background:**

Optimal management of locally recurrent prostate cancer after definitive radiation therapy is still challenging. With the development of highly accurate radiotherapy devices, prostate salvage re-irradiation might generate lower toxicity rates than classical salvage therapies. We retrospectively evaluated the toxicity and the feasibility of a prostate re-irradiation after definitive radiation therapy failure. Two modalities were investigated: high-dose-rate brachytherapy (HDRB) on whole prostate gland and focal stereotactic radiotherapy (SBRT) using CyberKnife® linac.

**Methods:**

Between 2011 and 2015, 28 patients with imaged and/or biopsy-proven intra-prostatic recurrence of cancer after definitive radiation therapy underwent a salvage re-irradiation using HDRB (*n* = 10) or focal SBRT (*n* = 18). The schedule of re-irradiation was 35 Gy in 5 fractions.

Biological response (defined as post-salvage radiation PSA variation) and biochemical no-evidence of disease (bNED) were evaluated in the whole cohort. For patients who had a positive biological response after salvage radiation, biochemical recurrence (BCR) and survival after salvage radiotherapy were evaluated. Post-salvage toxicities were assessed according to the Common Terminology Criteria for Adverse Events (CTCAE) v4.03 and were compared to baseline status.

**Results:**

Within a median follow-up of 22.5 months (IQR = 8–42), 9 (90%) patients experienced a positive biological response after salvage HDRB and 5 (50%) remained bNED at the end of the follow-up. Among patients who initially responded to salvage HDRB, the BCR rate was 44.4% after a median interval of 19.5 months (IQR = 11.5–26). Only one patient experienced a transient grade 3 urinary complication.

In the SBRT group, the median follow-up was 14.5 months (IQR = 7–23) and 10 (55.6%) out of the 18 patients remained bNED. Among the 15 patients who initially responded to salvage SBRT, 5 (33.3%) experienced a BCR. One patient experienced a transient grade 4 urinary complication.

At the end of the follow-up, all evaluated patients had a urinary status grade variation ≤ +1 grade. No grade 3–4 digestive toxicity was observed.

**Conclusions:**

Salvage prostate re-irradiation for locally recurrent cancer is feasible and generate low toxicities rates when using with HDRB or focal SBRT. However, further investigations are necessary to confirm these findings and to determine predictive features for patients who might benefit from such an approach.

## Introduction

Although external beam radiation therapy (EBRT) and brachytherapy are very efficient treatment modalities for localized prostate cancer, more than the half of the patients would experience a biochemical recurrence (BCR) within 10 years [1, 2]. Among these patients, about 20% would present a local recurrence for which most of the physicians would choose androgen-deprivation therapy (ADT) as the cornerstone of the therapy management [3]. But ADT remains a palliative treatment which significantly impacts quality of life [4]. Furthermore, tumor sensitivity to ADT is transient and cancer would eventually become castration-resistant. Therefore, preventing or delaying the introduction of ADT after PSA failure after initial treatment appears as a major challenge.

Local salvage procedures such as radical prostatectomy, high-intensity ultrasounds ablation (HIFU), cryosurgery or prostate re-irradiation [5–8] are therapy alternatives that can be offered to highly selected patients. Prostate re-irradiation was mainly done with low-dose-rate brachytherapy using seeds, with mixed results on efficacy and toxicity [5, 9, 10]. However, recent improvement in radiotherapy devices such as high-dose rate brachytherapy (HDRB) and stereotactic body radiotherapy (SBRT) allow to deliver higher ablative dose in a smaller target volume with better sparing of surrounding critical organs at risk [11, 12]. In spite of their encouraging results, very few patients benefit from these treatment options [3].

Actually, available data on post-radiation salvage HDRB and SBRT are very sparse and heterogeneous. Reported 2-year BCR-free survival are around 50% for both radiation modalities [13–20]. The aim of this study was to evaluate the clinical outcomes (efficacy and toxicity) of salvage HDRB or SBRT (CyberKnife®, Accuracy, Sunnyvale, CA, USA) in patients with post-radiation local recurrence of prostate cancer.

## Material and methods

### Patients’ selection

**Fig. 1:**
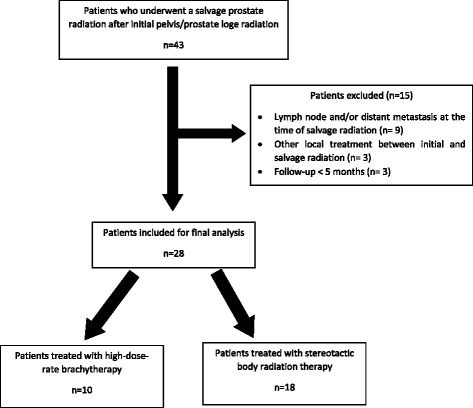
Patients’ flow chart

We retrospectively reviewed medical records of patients treated with salvage HDRB or CyberKnife® for a post-radiation prostate cancer local recurrence between 2011 and 2015 at Antoine-Lacassagne Cancer Center, Nice, France and at Clinique Hartmann, Levallois-Perret, France. All cases included in this study were discussed and approved by a multidisciplinary team. All patients provided a written informed consent for this institutions’ review boards-approved study. A total of 43 patients were reviewed. Patients with proven lymph node and/or distant metastasis at the time of salvage radiation (*n* = 9), those who underwent another local treatment between primary and salvage radiation (*n* = 3), and patients with a follow-up shorter than 5 months (*n* = 3) were excluded from the study, leaving 28 patients to be included for final analysis (Fig. 1).

### Follow-up after primary radiation

All patients experienced a BCR according to Phoenix criteria (PSA nadir + 2 ng/mL) or 1997 ASTRO criteria (three consecutive rises of PSA level above the nadir). Imaging investigations following BCR included pelvic and prostatic MRI (T2-weighted, diffusion-weighted and dynamic contrast-enhanced), [11C]-choline positron emission computed tomography (PET-CT) and thorax, abdominal and pelvic CT-scan. The indications of prostate biopsy or ADT were retained after multidisciplinary discussion of each case. Salvage re-irradiation was considered only in patients with a relapse delay > 24 months after initial radiation and a life-expectancy > 10 years. Prostate biopsies were required before considering local salvage therapy. However, in case of histological impossibility to determine a precise Gleason score but with pathological features of malignancy together with a body of evidence in favor of a local recurrence (PSA kinetic, prostate MRI and [11C]-PET-CT findings) patients remained eligible for salvage radiation. Post-radiation complications were recorded using the Common Terminology Criteria for Adverse Events (CTCAE v4.03) [21].

### Salvage radiation modalities

#### Stereotactic body radiation therapy protocol

Salvage SBRT was delivered with a CyberKnife® accelerator (Accuray, Sunnyvale, CA, USA). For tracking modality, at least three gold fiducials were placed in the prostate via trans-rectal ultrasonography (TRUS). Then, patients underwent a non-contrast-enhanced multi-slice CT scan (GE LightSpeed Scanner, GE Healthcare Diagnostic Imaging, Slough, UK) with 1.25 mm slice thickness. Patient immobilization during CT acquisition and treatment was obtained by a custom-build holding device. Prostate index tumor was defined by MRI and/or [11C]-choline PET and was considered as the gross tumor volume (GTV). The GTV was delineated by using image fusion of MRI and/or [11C]-choline PET with planning CT. Assuming that the clinical target volume (CTV) would be the GTV plus a 1-mm margin, the planning target volume (PTV) was delineated as the CTV plus a 1-mm margin leading to consider a focal SBRT. On each slice, contouring of organs at risk (rectum, bladder and femoral heads) were also performed. All plans were optimized using Multiplan® treatment planning system (Accuray, Sunnyvale, CA, USA). The PTV received 35 Gy in 5 fractions of 7 Gy each in 5 consecutive days within the 80% coverage dose. The dose constraints for organs at risk were applied according to the American Association of Physicists in Medicine recommendations [22].

### High dose rate brachytherapy protocol

The method of HDRB has been previously described [23]. Briefly, under general anesthesia and after urinary catheterization, 17 HDRB trans-perineal vectors (Sharp Needles™, Elekta AB, Stockholm, Sweden) were placed under TRUS guidance. The whole prostate gland and, if needed, the proximal part of the seminal vesicles were implanted using a dedicated perineal template. Due to vectors migration occurring after the implant [24], a new CT-scan was performed before each fraction allowing a specific re-planification and re-optimization of treatment plans. The CTV was defined as the outer contour of the prostate and included proximal part of the seminal vesicles if necessary. A total dose of 35 Gy was delivered in 5 fractions over 5 consecutive days. Urethra and rectum were delineated as organs at risk. According to recommendations of GEC/ESTRO [25], the following dose constrains for target volume were applied: the percentage of CTV receiving 100% of the prescribed isodose (V100) was ≥95%; V150 was limited to <30%, and V200 was limited to <12%. The dose non-homogeneity ratio (DNR) had to be <0.30. For the urethra, V115 was kept to <1% and for the rectum, V80 was kept to <1%. For both SBRT and HDRB, the doses received by organs at risk at initial treatment were not taken into account.

### Androgen deprivation therapy (ADT)

At the time of recurrence, ADT was dispensed to 2 (20.0%) patients that were further treated with salvage HDRB and one (10.0%) of them did not discontinue his treatment before salvage HDRB. In the SBRT group, 10 (55.6%) patients were receiving ADT before salvage treatment and for 3 (16.7%) of them ADT was not withdrawn after salvage SBRT.

### Follow-up after salvage radiation and endpoints’ definitions

After salvage re-irradiation, PSA level dosage and clinical assessment of toxicities according to the CTCAE v4.03 were recorded at 2 months after treatment, then every 3 months for a year, then every 6 months. For the purpose of this study, biochemical response was defined according to PSA level variation (ΔPSA) between pre-salvage and post-salvage re-irradiation: biochemical positive response was defined as a diminution of ΔPSA of more than 20%, biochemical failure corresponded to ΔPSA progressing of more than 20% and biochemical stabilization was defined between these two ranges.

In case of biochemical failure, a new [11C]-choline-PET-CT was performed to define local, regional or distant failure. A systemic treatment (ADT or chemotherapy) was introduced according to European Association of Urology guidelines [7]. For patients who experienced biochemical positive response or stabilization, post-salvage BCR was defined according to Phoenix criteria taking post-salvage PSA nadir level as a reference.

The primary endpoint was progression-free survival defined as biochemical no evidence of disease (bNED). For the group of patients who experienced a positive biochemical response or stabilization after salvage re-irradiation, other analyzed survival covariates were BCR-free survival, local recurrence-free survival, lymph-node recurrence-free survival, metastatic-free survival and systemic therapy-free survival. Because some patients experienced late effects of the primary irradiation at the time of BCR and in order to assess the specific effect of prostate re-irradiation on organs at risk (urethra and rectum), acute (<3 months) and late (>3 months) toxicities were expressed as grade variations compared to baseline status using CTCAE v4.03 [21].

### Statistical analyses

Continuous variables were expressed with median and interquartile range and were compared using the Kruskal-Wallis test. Categorical variables were compared with the *χ*2 test. Survival probabilities were estimated by the Kaplan-Meir method. Cases were censored at the last follow-up. A *p*-value < 0.05 was considered significant. Statistical analyses were carried out using Stata v11 (College Station, TX, United States).

## Results

### Patients’ characteristics

Finally, within a median follow-up of 15.5 months (IQR = 7–28), 28 patients were included in this study. Among them, 10 patients underwent HDRB while 18 patients underwent SBRT (Fig. 1). Table 1 summarizes patients’ characteristics at primary treatment and at recurrence.

**Table 1:** Patients’ characteristics

Characteristics	High-dose-rate brachytherapy (*n* = 10)		Stereotactic body radiation therapy (*n* = 18)		*p* value	*p* value
	Primary treatment (%)	Recurrence (%)	Primary treatment (%)	Recurrence (%)	Primary	Recurrence
Age (years, median, IQR)	63 (58–68)	69 (65–77)	62 (58–66)	69 (64–75)	0.87	0.85
PSA at the time of treatment (ng/mL, median, IQR)	26 (8.6–47)	4.37 (2.01–4.76)	6.6 (5.7–9.2)	4.5 (3.0–6.3)	0.0046	0.43
Initial T stage		-		-	0.005	
T1c	2 (20.0)		12 (66.7)			
T2a	2 (20.0)		2 (11.1)			
T3	6 (60.0)		2 (11.1)			
Not evaluated	-		2 (11.1)			
Initial Gleason sum					0.04	0.71
Gleason 6 or less	2 (20.0)	1 (10.0)	13 (72.2)	2 (11.1)		
Gleason 7	4 (40.0)	2 (20.0)	4 (22.2)	2 (11.1)		
Gleason 8	4 (40.0)	1 (10.0)	1 (5.6)	1 (5.6)		
Gleason 9	-	2 (20.0)	-	2 (11.1)		
Not evaluated	-	4 (40.0)	-	11 (61.1)		
D’Amico risk group					0.006	
Low	1 (10.0)	-	10 (55.6)	-		
Intermediate	2 (20.0)		5 (27.8)			
High	7 (70.0)		2 (11.1)			
Not evaluated	-		1 (5.6)			
Tumor localization					0.21	0.019
Apex	-	1 (10.0)	-			
Median	-	-	2 (11.1)	2 (11.1)		
Basis	-	3 (30.0)	1 (5.6)	1 (5.6)		
Seminal vesical	1 (10.0)	-	-	7 (38.9)		
More than 1 location	1 (10.0)	6 (60.0)	3 (16.7)	3 (16.7)		
Not evaluated	8 (80.0)	1 (10.0)	12 (66.7)	4 (22.2)		
Initial ADT duration		-		-	0.0031	
Short (6 months)	2 (20.0)		7 (38.9)			
Long (24–36 months)	5 (50.0)		1 (5.6)			
No ADT	3 (30.0)		10 (55.6)			
ADT at recurrence	-		-			0.05
Yes		2 (20.0)		10 (55.6)		
No		8 (80.0)		8 (44.4)		
ADT duration (months, median, IQR)	-	63.5 (48–79)	-	15 (6–21)		0.028
Initial radiation modality		-		-	<0.001	
LDR brachytherapy	1 (10.0)		15 (83.3)			
EBRT w/o pelvic radiation	5 (50.0)		3 (16.7)-			
EBRT with pelvic radiation	4 (40.0)					
Prostate volume (cc, median, IQR)	33.5 (32–35)	35 (20–50)	35 (25–44.5)	26 (22.5–28.5)	0.78	0.59
PSA nadir (ng/mL, median, IQR)	0.065 (0.01–0.2)	0.66 (0.23–1.13)	0.58 (0.34–1.05)	0.89 (0.29–1.4)	0.0034	0.97
Time to PSA nadir (months, median, IQR)	21 (16–30)	6 (5–8)	28 (11–35)	7.5 (4.5–10)	0.79	0.89
Time to biological recurrence (months, median, IQR)	69 (55–85)	13 (10–26)	49 (37–70)	5.5 (4–6)	0.11	0.0347
Time to salvage treatment (months, median, IQR)	86.5 (66–108)	-	77 (64–92)	-	0.26	

Note: *PSA* prostate specific antigen, *ADT* androgen deprivation therapy, *IQR* interquartile range, *LDR* low-dose-rate, *EBRT* external beam radiotherapy

Patients treated with salvage SBRT had mainly received low-dose-rate brachytherapy (83.3%) for low-risk group of D’Amico initial prostate cancer (55.6%), whereas the HDRB group was mainly constituted of patients with initial high-risk disease (70.0%) treated with EBRT (90.0%) (*p* = 0.006 and *p* < 0.001, respectively). The median time to BCR was 69 months (IQR = 55–85) and 49 months (IQR = 37–70) for HDRB and SBRT groups, respectively (*p* = 0.11). There was no significant difference of delay between primary and salvage radiation in both groups (86.5 months (IQR = 66–108) vs. 77 months (IQR = 64–92) for HDRB and SBRT respectively, *p* = 0.26).

### Oncologic outcomes after salvage high-dose-rate brachytherapy

**Fig. 2:**
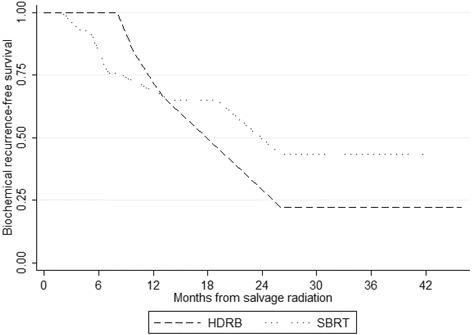
Biochemical recurrence-free survival after salvage high-dose-rate brachytherapy (*n* = 10) and stereotactic body radiation therapy (*n* = 18)

Ten patients underwent salvage HDRB within a median follow-up of 22.5 months (IQR = 8–42) and 5 (50%) remained bNED at the end of the follow-up. Dosimetric features of HDRB are resumed in Table 2a. The median PSA level at the time of salvage HDRB was 4.42 ng/mL (IQR = 2.01–6.7). Nine (90%) patients had a biochemical positive response with a median ΔPSA of -2.81 ng/mL (IQR = -5.6;-1.16) and 1 (10%) patient experienced a biochemical progression despite salvage HDRB. For patients who responded to salvage HDRB (*n* = 9, 90%), median PSA nadir was 0.66 ng/mL (IQR = 0.23–1.13) and was reached within 6 months (IQR = 5–8) after salvage radiation. Among patients who initially experienced biochemical positive response to HDRB (*n* = 9, 90%), 4 (44.4%) of them experienced BCR within 19.5 months (IQR = 11.5–26) after salvage therapy (Fig. 2). Three (33.3%) patients experienced image-proven loco-regional disease recurrence within 27 months (IQR = 14–36) and 2 patients (22.2%) experienced metastatic disease progression within a median time interval of 25 months (IQR = 14–36). When including patients who did not interrupted their ADT, median time before the introduction of systemic treatment was 14 months (IQR = 6–27). With a follow-up of 21 months, the patient who experienced immediate HDRB biochemical failure did not developed extra-prostatic disease progression after introduction of ADT.

**Table 2:** Dosimetric features for high-dose-rate brachytherapy (a) and cyberknife (b)

a			
Parameter	Value		
CTV	35 (25–43)		
D90	106 (106–108)		
D100	81 (77–85)		
V100	97 (96–98)		
V150	24 (22–32)		
V200	8 (7–10)		
D2 rectum	57 (50–69)		
D2 urethra	81 (76–85)		
DHI	0.27 (0.22–0.33)		
b			
Region of interest	Mean (cGy)	Min (cGy)	Max (cGy)
GTV	4027	3462	4375
PTV	3936	3134	4375
Bladder	862	161	3680
Rectum	727	102	3561
Right femoral head	371	145	894
Left femoral head	428	139	1075

Note: *CTV* clinical target volume, *D90* dose delivered to 90% of the CTV, *D100* dose delivered to 100% of the CTV, *V100* volume receiving 100% of the prescribed dose, *V150* volume receiving 150% of the prescribed dose, *V200* volume receiving 200% of the prescribed dose, *D2 rectum* dose delivered to 2% of the rectum volume, *D2 urethra* dose delivered to 2% of the urethra volume, *DHI* dose non-homogeneity Index: V150/V100

Note: *GTV* gross target volume, *PTV* planning target volume

### Oncologic outcomes after salvage stereotactic body radiation therapy

Within a median follow-up of 14.5 months (IQR = 7–23), 10 (55.6%) out of the 18 patients who underwent salvage SBRT remained bNED. SBRT dosimetric characteristics are resumed in Table 2b. The median PSA level at the time of salvage SBRT was 4.5 ng/ml (IQR = 3.0–5.3). Thirteen patients (72.2%) presented a regression of PSA level after salvage SBRT with a median ΔPSA of -3.2 ng/mL (IQR = -5.34; -1.8), 2 patients (11.1%) had a PSA level stabilization and 3 (16.7%) patients experienced post-SBRT biochemical failure. When considering patients who had a positive response or stabilization (*n* = 15), nadir PSA level was 1.00 ng/mL (IQR = 0.42–2.4) and was reached within 7.5 months (IQR = 4.5–10) after salvage SBRT. Five patients (33.3%) experienced BCR within a median follow-up of 7 months (IQR = 4–7) (Fig. 2). Among them, 3 patients (20.0%) experienced loco-regional disease recurrence within 5 months (IQR = 4.9–9.5) and 1 (6.7%) had a metastatic progression of his disease. All the 3 patients who experienced a post-SBRT biochemical failure developed an extra-prostatic evolution despite the introduction of ADT.

### Toxicities after salvage radiation

Toxicities following salvage radiation are summarized in Tables 3.

**Table 3:** Complication grades and grade variations following salvage high-dose-rate brachytherapy (a, b) and stereotactic body radiation therapy (c, d) using CTCAE v4.03

a				
HDRB	At baseline	Maximal acute toxicity	Maximal late toxicity	At last follow-up
Urinary complication (n, %)				
No complication	5 (50.0)	-	2 (20.0)	2 (20.0)
Grade 1	4 (40.0)	2 (20.0)	-	3 (30.0)
Grade 2	1 (10.0)	7 (70.0)	6 (60.0)	5 (50.0)
Grade 3	-	-	1 (10.0)	-
Grade 4	-	-	-	-
Not evaluated		1 (10.0)	1 (10.0)	-
Digestive complication (n, %)				
No complication	7 (70.0)	8 (80.0)	7 (70.0)	10 (100.0)
Grade 1	3 (30.0)	1 (10.0)	2 (20.0)	-
Grade 2	-	-	-	-
Grade 3	-	-	-	-
Grade 4	-	-	-	-
Not evaluated	-	1 (10.0)	1 (10.0)	-
b				
HDRB	At 3 months		At last follow-up	
	n	%	n	%
Urinary complication				
No modification	2	20.0	4	40.0
+ 1 grade	3	30.0	5	50.0
+ 2 grades	4	40.0	1	10.0
Not evaluated	1	10.0	-	-
Digestive complication				
No modification	9	90.0%	10	100
+ 1 grade	-	-	-	-
+ 2 grades	-	-	-	-
Not evaluated	1	10.0%	-	-
c				
SBRT	At baseline	Maximal acute toxicity	Maximal late toxicity	At last follow-up
Urinary complication (n, %)				
No complication	8 (44.4)	2 (11.1)	6 (33.3)	4 (22.2)
Grade 1	5 (27.8)	5 (27.8)	4 (22.2)	6 (33.3)
Grade 2	3 (16.7)	2 (11.1)	1 (5.6)	3 (16.7)
Grade 3	1 (5.6)	-	-	-
Grade 4	-	-	1 (5.6)	-
Not evaluated	1 (5.6)	9 (50.0)	6 (33.3)	5 (27.8)
Digestive complication (n, %)				
No complication	13 (72.2)	5 (27.8)	9 (50.0)	10 (55.6)
Grade 1	1 (5.6)	1 (5.6)	-	1 (5.6)
Grade 2	2 (11.1)	2 (11.1)	1 (5.6)	2 (11.1)
Grade 3	-	-	-	-
Grade 4	-	-	-	-
Not evaluated	2 (11.1)	10 (55.6)	8 (44.4)	5 (27.8)
d				
SBRT	At 3 months		At last follow-up	
	n	%	n	%
Urinary complication				
No modification	4	22.3	12	66.7
+ 1 grade	4	22.2	1	5.6
+ 2 grades	1	5.6	-	-
Not evaluated	9	50.0	5	27.8
Digestive complication				
No modification	7	38.9	12	66.7
+ 1 grade	-	-	-	-
+ 2 grades	1	5.6	-	-
Not evaluated	10	55.6	6	33.3

During the course of HDRB and within 3 months thereafter, most patients experienced grade 1–2 toxicities (Table 3a). When comparing to baseline, 2 patients (20%) had no modification of their acute urinary status, 3 (30%) experienced an increase of +1 grade and 4 (40%) of +2 grades.

When considering late toxicities, 5 patients (50%) had a urinary condition worsen of +1 grade and 1 patient (10%) had a +2 grade worse condition (Table 3b). One (10%) patient experienced a grade 3 hematuria 12 months after salvage HDRB, treated by hyperbaric chamber. No grade 3–4 digestive toxicity occurred during the follow-up.

After salvage SBRT, no patient experienced grade 3–4 digestive toxicity (Table 3c). One patient (5.6%) experienced a septic shock following a prostatitis complicated by a histology-proven prostate necrosis 12 months after salvage SBRT. This patient already had a history of prostatitis and underwent several transurethral resections of the prostate for benign prostatic obstruction. At last follow-up, 12 patients (66.7%) had no modification of their urinary and digestive status compared to baseline (Table 3d).

## Discussion

Despite its wide range of side effects and the absence of curative perspective, ADT remains the most chosen option in case of local recurrence of prostate cancer after definitive radiation therapy [3, 4]. However, international guidelines also consider the possibility to offer a local salvage therapy to some patients who meet the criteria of a strictly intra-prostatic disease [7]. For these highly selected patients (PSA < 10 ng/ml, Gleason score ≤ 7, PSA doubling time ≥ 16 months and life expectancy > 10 years), a salvage treatment modality can be proposed in order to control the local disease, and delay, or better still, suppress the indication of ADT.

Due to the reluctance of physicians to intervene on potentially vulnerable tissues, local salvage options are hardly ever proposed to patients [3]. In this context, salvage prostate re-irradiation can only be considered and investigated if this option is associated with low toxicity rates.

Therefore, in this retrospective study, we evaluated the toxicity and the feasibility of a prostate re-irradiation after definitive radiation therapy failure. Two modalities were investigated: HDRB and SBRT using CyberKnife®.

Available data on salvage re-irradiation with HDRB are sparse and were mainly limited by their heterogeneity for both the type of patients included and the radiation protocols used [13–16, 26]. Furthermore, they were often conducted on small cohorts. However, reported 2-year bNED or BCR-free survivals ranged from 50 to 89%. To date, only one prospective study is available. In this phase II study, Yamada et al. treated 42 patients with a total dose of 32 Gy in 4 fractions [17]. Within a median follow-up of 36 months, 68.5% remained free of BCR.

After definitive radiation therapy for the primary prostate cancer, late grade 1–2 and 3–4 urinary complications are about 17% and 3%, respectively. For digestive toxicity, 15% of the patients would experience grade 1–2 complications and 2% of them would experience grade 3–4 adverse events [27]. Logically, at the time of local salvage re-irradiation on potentially weakened tissues, higher morbidity rates are expected whatever the chosen option is. This is also true for salvage prostatectomy which is associated with the highest rates of complications, even if it remains the most efficient option for oncologic outcomes [28–30]. Whole prostate gland cryosurgery and low-dose-rate brachytherapy with seeds have similar survival rates with HDRB, but are associated with poorer functional outcomes [28, 30]. Indeed, the most recently published protocols of HDRB were associated with a grade 3 urinary complication rate between 2 and 20%, with almost none severe digestive complications [13, 15–17, 26]. With a bNED rate of 50% after 22.5 months of median follow-up and very low severe toxicity rates, our results on salvage HDRB are very encouraging and are comparable to published data. In this context, it is worth proposing a prostate re-irradiation to some highly selected patients.

To date, only few studies have been published on salvage re-irradiation using a CyberKnife® linac. Vavassori et al. published the first results on prostate re-irradiation using this system. In this preliminary retrospective case series including 6 patients treated with a total dose of 30 Gy in 5 fractions on the whole prostate gland, 2 patients remained bNED after 11.2 months of median follow-up [18]. Since then, Fuller et al. published a retrospective study including 29 patients treated with a total dose of 34 Gy in 5 fractions within a median follow-up of 24 months. They reached an actuarial 2-year BCR-free survival of 82% with only 7% of grade 3–4 urinary toxicity and no severe digestive toxicity [20].

Our approach for salvage SBRT was slightly different as we used focal re-irradiation instead of whole gland treatment. We reached a bNED rate of 55.6% at 14.5 months of median follow-up and a BCR-free survival of 66.7% with reasonable induced toxicity. Janoray et al. recently published a report on focal salvage SBRT using CyberKnife® after EBRT failure [31]. In this retrospective study including 11 patients, they reached a biochemical response rate of 82% (9 out of 11 patients). At twelve months of follow-up, 6 patients were free of disease recurrence. Our results on survival outcomes are similar to those obtained by other focal salvage therapies such as cryosurgery, HIFU or low-dose-rate brachytherapy. Reported 1-year BCR-free survival ranged from 69 to 100% and dropped to 49–100% at 2-year [32]. Severe reported urinary and digestive toxicities ranged from 0 to 33.3%. In our study, most of the patients had transient low-grade complications and returned to baseline at the last follow-up.

Therefore, both salvage HDRB and focal SBRT appear as suitable options to delay the introduction of ADT in some highly selected patients, without exposing patients to an unreasonable risk of complication. But the scope of our results is limited by the retrospective setting of our study, its small cohort and its short follow-up that make it not calibrated to establish cancer-specific and overall survival rates. Furthermore, even if few patients did not discontinue their ADT after salvage radiation, this fact constitutes a bias for the interpretation of survival outcomes. Indeed, the heterogeneous population including both hormone-refractory and hormone-naïve prostate cancers together with both low- and high-grade cancers probably impacted oncologic outcomes. Therefore, the determination of predictive factors for radiation sensitive cancers would help to refine the selection of patients who could benefit from a salvage prostate re-irradiation. These questions have been discussed during the Delphi conference of consensus on salvage brachytherapy [33], but many crucial questions such as maximal PSA value at the time of salvage brachytherapy, prostate gland volume to treat (whole gland, half or focal) or radiation doses did not reach a consensus.

The choice to perform a focal radiation with CyberKnife® has been encouraged by oncologic results of focal cryosurgery and HIFU which were comparable to whole gland treatment with less toxicity [32]. But as all focal treatments, the main limitation remains in the definition of the target volume. Even if we used fusion of MRI and [11C]-choline-PET, we can’t guaranty that the visualized lesions are the only cancerous spots in the prostate.

But this study has also strengths: our hypo-fractionation scheme for HDRB allowed, contrarily to most of the studies published on this topic, to treat patients within 5 consecutive days with only one implant. This report on salvage focal CyberKnife® shows promising results that would worth to be confirmed in a larger and prospective manner.

## Conclusion

For highly selected patients, salvage prostate re-irradiation using HDRB or focal SBRT are suitable options to treat local recurrence of cancer after definitive radiation therapy. The low toxicity rates associated with both of these techniques are encouraging proposing these options to a well-defined group of patients, without exposing them to an unreasonable risk. However, further investigations are needed to confirm these findings and to define selection criteria for patients who could benefit from such approaches.

## Abbreviations

ADT: Androgen-deprivation therapy
BCR: Biochemical recurrence
bNED: Biological non-evidence of disease
CT: Computerized tomography
CTCAE: Common Terminology Criteria for Adverse Events
CTV: Clinical target volume
EBRT: External beam therapy
GTV: Gross target volume
HDRB: High-dose rate brachytherapy
HIFU: High-intensity ultrasounds ablation
IQR: Interquartile range
MRI: Magnetic resonance imaging
PET: Positron emission computed tomography
PSA: Prostate specific antigen
PTV: Planning target volume
SBRT: Stereotactic body radiotherapy
TRUS: Trans-rectal ultrasonography
